# Mode I Interlaminar Fracture of Glass/Epoxy Unidirectional Laminates. Part II: Numerical Analysis

**DOI:** 10.3390/ma12101604

**Published:** 2019-05-16

**Authors:** A. Gliszczynski, S. Samborski, N. Wiacek, J. Rzeczkowski

**Affiliations:** 1 Lodz University of Technology, Faculty of Mechanical Engineering, Department of Strength of Material, 90-924 Lodz, Stefanowskiego 1/15, Poland; nina.wiacek@gmail.com; 2 Lublin University of Technology, Faculty of Mechanical Engineering, Department of Applied Mechanics, 20-618 Lublin, Nadbystrzycka 36, Poland; s.samborski@pollub.pl (S.S.); kubarzeczkowski@op.pl (J.R.)

**Keywords:** DCB, Mode I, GFRP, bilinear law, exponential law, VCCT, CZM

## Abstract

The paper deals with numerical analysis of double cantilever beam (DCB) predefined to Mode I Interlaminar Fracture Tests of GRFP unidirectional laminates. The numerical analyses were performed in the ANSYS^®^ program based on the finite element. In geometrically nonlinear analysis, two algorithms, responsible for initiation and propagation of delamination front, were applied: Virtual Crack Closure Technique (VCCT) and Cohesive zone method (CZM). Due to the unidirectional arrangement of layers of the laminate, the problem of DCB test was solved with the use of one- and three-dimensional models with the implementation of linear interface element and contact element. The present study highlights the limitations of existing formulae used to reliably reflect the behavior of DCB. The use of three-dimensional models allowed confirming the curved shape of the delamination front observed in experimental studies. The application of the VCCT in the three-dimensional model led to an underestimation of the global response (force–opening displacement curve) recorded during numerical DCB test.

## 1. Introduction

Nowadays, composite materials are used in many industries, such as the aviation industry [1], aerospace engineering [2], civil engineering [3,4,5] and the automotive industry [6]. The most common damage form of composite materials is delamination [7] initiated by notch, ply drop, bonded or bolted joint, free edges, buckling or pressure [8]. From the numerical point of view, crack growth is a phenomenon in which two surfaces are separated from each other, or material is progressively damaged under external loading, and in relation to composite materials can be modeled by the application of: the virtual crack-closure technique (VCCT) [9,10], the cohesive zone method (CZM) [11,12] and the extended finite element method (XFEM) [13,14,15,16].

Delamination phenomenon, limiting the toughness and ductility of multi-phase composite materials, has motivated considerable research on the failure of the interfaces. The importance of the cohesive-zone approach to analyze localization and failure in engineering materials was emphasized by de Borst [17]. The author discussed various ways to incorporate the cohesive-zone methodology in computational methods and stated that the proper representation of the discrete character of cohesive-zone formulations that avoids any mesh bias can be obtained elegantly when exploiting the partition-of-unity property of finite element shape functions. Harper and Hallett [18] highlighted the limitations of existing formulae used to predict numerical cohesive zone length and demonstrated modifications necessary for improved accuracy. They also provided the clarification regarding the minimum number of interface elements within the cohesive zone. Herráez et al. [19] developed a numerical framework to obtain the crack resistance curve and its corresponding softening law for fracture analysis in composite materials under small scale bridging. A methodology to determine the constitutive parameters for the simulation of progressive delamination, taking into account the size of a cohesive finite element and the length of the cohesive zone and ensuring the correct dissipation of energy, was proposed by Turon et al. [20]. They provided a closed-form expression for estimating the minimum penalty stiffness necessary for the constitutive equation of a cohesive finite element and showed that the resulting constitutive law allows the use of coarser finite element meshes than is usually admissible. Sensitivity studies of the double cantilever beam (DCB) test, based on kriging analysis of FE models, were conducted by Gustafson and Waas [21]. The authors stated that in relation to opening mode the DCB model is only sensitive to Mode I critical energy release rate; therefore, the DCB test is useful as an independent physical test to determine fracture toughness for cohesive zone FE models. Additionally, no model investigated by the authors was significantly affected by the value of critical traction stress which allowed them to conclude that critical normal stress in Mode I can be determined independently in physical tests. Agrawal and Karlsson [22] reviewed, unified and extended work pertaining to evaluating mode mixity of interfacial fracture utilizing the VCCT. Although the XFEM method was not analyzed in this study, it should be noted that it also allows achieving valuable results. A novel set of enrichment functions within the framework of the extended finite element method (XFEM) was proposed by Wang and Waisman [15] for linear elastic fracture analysis of interface cracks in bimaterials. Wang et al. [16] presented a high-order XFEM with a set of material-dependent enrichment functions for the linear elastic analysis of bimaterial interface cracks. The authors examined the effect of enrichment order, mesh refinement, bimaterial mismatch, crack tip position, and integration limit of Irwin’s integral and proved that the proposed approach does not require special auxiliary fields for curved interface cracks.

As the VCCT and the CZM for analyzing composite delamination are incorporated into finite element codes, the need for comparison and benchmarking becomes important, inasmuch as each code requires specific input parameters unique to its implementation. These parameters are unique to the numerical approach chosen and do not reflect real physical differences in delamination behavior. An approach for assessing the Mode I delamination propagation capabilities in commercial finite element codes under static loading was presented and demonstrated in ABAQUS/Standard^®^ [23] as well as MD Nastran™ and Marc™ [24]. Within this article, benchmark studies in ANSYS^®^ of two- and three-dimensional DCB model with implementation of contact and cohesive elements, where the crack growth phenomenon was modeled using the VCCT and the CZM with the implementation of the bilinear and the exponential delamination law, were performed. The results presented will aid the application of mesh design techniques that preserve numerical accuracy, while minimizing computational expense. In addition, the functionalities of the CZM and VCCT methods are presented, the differences between the techniques used were explained, and the experimentally observed shape of the propagating crack front propagating for unidirectional composite was confirmed. The application of the VCCT in the three-dimensional model leads to an underestimation of the global response (force–opening displacement curve) recorded during numerical DCB test. In addition, it was noted that scratches left by the propagating front of the delamination correspond with the results of the ultrasonic C-scan image of DCB specimen after fatigue precracking obtained by Czabaj and Ratcliffe [25].

## 2. Crack-Growth Simulation and Interface Delamination

Crack-growth simulation in homogenous and composite structures is of interest because of the need for structural integrity assessments. Pre-existing cracks may propagate when certain loading conditions are reached or when certain localized conditions are met. Cracks may also propagate along the interface between the layers of a composite structure (interfacial delamination). Several fracture mechanics methods are available to simulate crack growth along a predefined path or along an arbitrary path.

For crack growth along the interfaces, the VCCT-based crack-growth simulation has become a widely used technique for simulation of interface delamination of laminate composite. The technique is also well suited for modeling the fracture process in a homogeneous medium, as fracture can be considered a separation process between two surfaces.

Alternatively, the cohesive zone model to simulate interface delamination and other fracture phenomena can be used. This approach introduces failure mechanisms by using the hardening–softening relationships between the separations and incorporating the corresponding tractions across the interface. Interface delamination can be modeled with either interface elements or contact elements (debonding).

### 2.1. Virtual Crack Closure Technique (VCCT)

The virtual crack closure technique (VCCT) was initially developed to calculate the energy-release rate of a cracked body [9]. It has since been widely used in the interfacial crack-growth simulation of laminate composites, with the assumption that crack growth is always along a predefined path, specifically the interfaces. Generally, the VCCT is based on the assumption that the energy needed to separate a surface is the same as the energy needed to close the same surface. The applied method uses the modified crack-closure method (a VCCT-based method) and assumes further that stress states around the crack tip do not change significantly when the crack grows by a small amount (Δa).

**Figure 1:**
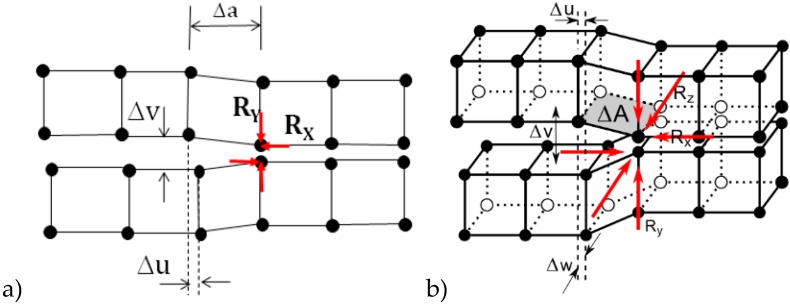
2-D (**a**); and 3-D (**b**) crack geometry schematic.

For 2-D and 3-D crack geometry with a low-order element mesh, the energy-release rates are defined as:

(1) $$G_{I}=-\frac{1}{2\Delta A}R_{Y}\Delta v$$

(2) $$G_{II}=-\frac{1}{2\Delta A}R_{X}\Delta u$$

(3) $$G_{III}=-\frac{1}{2\Delta A}R_{Z}\Delta w$$

where G_I_, G_II_, and G_III_ are the Modes I, II and III energy-release rate, respectively; Δu, Δv, and Δw are the relative displacement between the top and bottom nodes of the crack face in local coordinates x, y and z, respectively; R_X_, R_Y_, and R_Z_ are the reaction forces at the crack-tip node; and ΔA is the crack-extension (2D) or crack-extension area (3D) (cf. Figure 1).

To model the crack growth, it is necessary to define a fracture criterion for crack onset and the subsequent crack growth. For linear elastic fracture mechanics (LEFM) applications, the fracture criterion is generally assumed to be a function of critical energy-release rates. In the analyzed cases the linear function of fundamental modes was assumed in the following form:

(4) $$f=\frac{G_{I}}{G_{I}^{c}}+\frac{G_{II}}{G_{II}^{c}}+\frac{G_{III}}{G_{III}^{c}}$$

where $G_{I}^{c}$, $G_{II}^{c}$, $G_{III}^{c}$ are the Modes I, II and III critical energy-release rate, respectively.

In the case of unidirectional laminates loaded according to Mode I, the application of the more complicated fracture criteria (e.g., Benzeggagh-Kenane (B-K) criterion [26]) is unfounded. Due to the negligible values of G_II_ and G_III_ energies in Mode I, most of the fracture criteria are reduced; similar to the applied linear law, they reduce to formula:$f\approx G_{I}/G_{I}^{c}$. It should be noted that the VCCT method has a limitation (due to the calculation method of G_i_, the VCCT requires prior definition of the crack front) that prevents its application to computation of certain phenomena, e.g., a propagation of delamination during the impact.

### 2.2. Cohesive Zone Method

**Figure 2:**
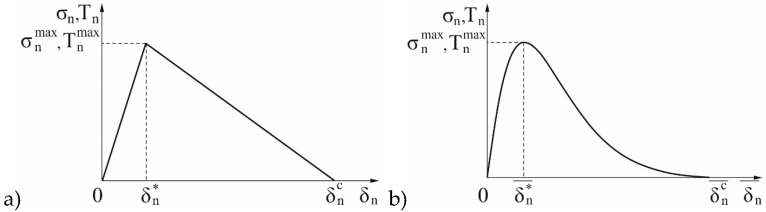
Bilinear (**a**); and exponential (**b**) law of delamination.

The cohesive zone material (CZM) model directly introduces fracture mechanism by adopting softening relationships between tractions and the separations, which in turn introduce a critical fracture energy that is also the energy required to break apart the interface surfaces. The interface surfaces of the materials can be represented by a special set of interface elements or contact elements. The CZM model consists of a constitutive relation between the traction T acting on the interface and the corresponding interfacial separation δ (displacement jump across the interface). The definitions of traction and separation depend on the element and the material model. In the conducted numerical research, exponential and bilinear CZM models were used (cf. Figure 2).

An exponential form of the CZM model, originally proposed by Xu and Needleman [11], uses a surface potential:

(5) $$\phi \left(\delta \right)=e\sigma_{\max}\bar{\delta_{n}}\left[1+\left(1+\Delta_{n}\right)e^{-\Delta_{n}}e^{-\Delta_{t}^{2}}\right]$$

where Φ(δ) is the surface potential; σ_max_ is the maximum normal traction; δ, $\delta_{n}^{}=\vec{n}\delta$, and $\delta_{t}^{}=\vec{t}\delta$ are the interfacial, normal and shear separation, respectively; $\bar{\delta_{n}^{}}$ ($\bar{\delta_{t}^{}}$) is the normal (shear) separation across the interface where the maximum normal (shear) traction is attained; $\Delta_{n}=\frac{\delta_{n}}{\bar{\delta_{n}}}$; and $\Delta_{t}=\frac{\delta_{t}}{\bar{\delta_{t}}}$.

The normal (Equation (6)) and shear (Equation (7)) traction are defined as:

(6) $$T_{n}=\frac{\partial \phi \left(\delta \right)}{\partial \phi \left(\delta_{n}\right)}=e\sigma_{\max}\Delta_{n}e^{-\Delta_{n}}e^{-\Delta_{t}^{2}}$$

(7) $$T_{t}=\frac{\partial \phi \left(\delta \right)}{\partial \phi \left(\delta_{t}\right)}=2e\sigma_{\max}\frac{\bar{\delta_{n}}}{\bar{\delta_{t}}}\Delta_{t}\left(1+\Delta_{n}\right)e^{-\Delta_{n}}e^{-\Delta_{t}^{2}}$$

The applied bilinear CZM model assumes that the separation of the material interfaces is dominated by the displacement jump normal to the interface (Mode I Dominated Bilinear CZM Model). A bilinear form of the CZM model, proposed by Alfano and Crisfield [12], defines the relation between normal (shear) cohesive traction T_n_ (T_t_) and normal (tangential) displacement jump δ_n_ (δ_t_) in the following forms:

(8) $$T_{n}=K_{n}\delta_{n}(1-D_{n})$$

(9) $$T_{t}=K_{t}\delta_{t}(1-D_{n})$$

where T_n_ (T_t_) is the normal (shear) cohesive traction $T_{n}^{\max}/\delta_{n}^{*}$, ($T_{t}^{\max}/\delta_{t}^{*}$); K_n_ (K_t_) is the normal (tangential) cohesive stiffness; δ_n_ (δ_t_) is the normal (tangential) displacement jump; $\delta_{n}^{*}$($\delta_{t}^{*}$) is the normal (tangential) displacement jump at maximum normal (shear) cohesive traction; $\delta_{n}^{c}$ ($\delta_{t}^{c}$) is the normal displacement jump at the completion of debonding; and $D_{n}$ is the damage parameter.

The damage parameter associated with Mode I dominated bilinear cohesive law is defined as:

(10) $$D_{n}=\{\begin{array}{cc} 0 & \delta_{n}^{\max}\leq \delta_{n}^{*}\\ \left(\frac{\delta_{n}^{\max}-\delta_{n}^{*}}{\delta_{n}^{\max}}\right)\left(\frac{\delta_{n}^{c}}{\delta_{n}^{c}-\delta_{n}^{*}}\right) & \delta_{n}^{*}<\delta_{n}^{\max}<\delta_{n}^{c}\\ 1 & \delta_{n}^{\max}<\delta_{n}^{c} \end{array}$$

For Mode I dominated cohesive law, the tangential cohesive traction and tangential displacement jump behavior is assumed to follow the normal cohesive traction and normal displacement jump behavior.

The effective elastic properties of the composite will not be affected by the cohesive surface whenever E_3_ << Kt. Thus, according to the recommendation of Turon et al. [20], the stiffness of the cohesive zone model can be estimated as Equation (11):

(11) $$K=\frac{\alpha E_{3}}{t}$$

where α is a parameter much larger than 1 (α >>1) and E_3_ is the through-the-thickness Young’s modulus of the material and for a transversely isotropic material can be assumed as equal to E_2_. It should be kept in mind that large values of the interface stiffness may cause numerical problems, such as spurious oscillations of the tractions [27]. Thus, the interface stiffness should be large enough to provide a reasonable stiffness but small enough to reduce the risk of numerical problems such as spurious oscillations of the tractions in an element. For values of α greater than 50, the loss of stiffness due to the presence of the interface is less than 2%, which is sufficiently accurate for most problems.

## 3. Analytical Solution for the Mode I DCB Test

In the international literature, plenty of analytical research devoted to Mode I investigation can be found. Multiple data reduction methods based on Linear Elastic Fracture Mechanics (LEFM) have been developed and can be categorized as: the compliance calibration methods based on experiments defining the relationship of the compliance versus the crack length [28,29]; the compliance calibration methods derived from the classical and modified beam theory [30,31]; an approach determining the energy release rate from the ratio of change in strain energy to the change in crack area [32]; and a reduction method using the J-integral as a fracture parameter [33]. The expressions for critical energy release rate used in this work are based on the work of Williams [30,34] and Hashemi et al. [31] leading to Equation (12). The necessary force P for crack propagation can be written as follows:

(12) $$P=\sqrt{\frac{G_{I}^{c}b^{2}h^{3}E_{1}}{12{\left(a+\chi h\right)}^{2}}}$$

where b is the width of the specimen (mm); h is the half of the thickness of the specimen (mm); E_1_ is the longitudinal Young’s modulus (MPa); Χh is the compensation term for the crack tip rotation effect (mm); and A is the length of the delamination (mm).

The compensation term responsible for the crack tip rotation effect can be determined directly from the compliance calibration curve. Nevertheless, the χ coefficient can be defined analytically as:

(13) $$\chi =\sqrt{\frac{E_{11}}{11G_{12}}\left[3-2{\left(\frac{\Gamma }{1+\Gamma }\right)}^{2}\right]}$$

where

(14) $$\Gamma =1.18\sqrt{\frac{E_{11}E_{22}}{G_{12}}}$$

The corresponding displacement δ for crack propagation is given by:

(15) $$\delta =P\frac{8{\left(a+\chi h\right)}^{3}}{bh^{3}E_{1}}$$

Eliminating (a + χh) in Equations (12) and (15), one obtains:

(16) $$P=\sqrt{8}{\left(\frac{G_{I}^{c}}{12}\right)}^{\frac{3}{4}}\frac{1}{\sqrt{\delta }}b{\left(h^{3}E_{1}\right)}^{\frac{1}{4}}$$

## 4. FE Models with the Assumed Boundary Conditions

**Figure 3:**
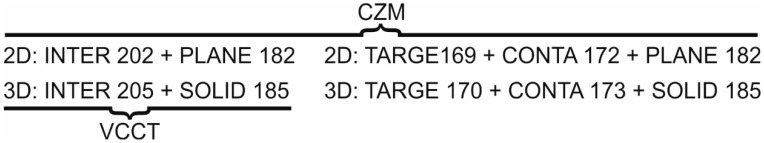
Finite elements used for numerical analyses [35].

**Figure 4:**
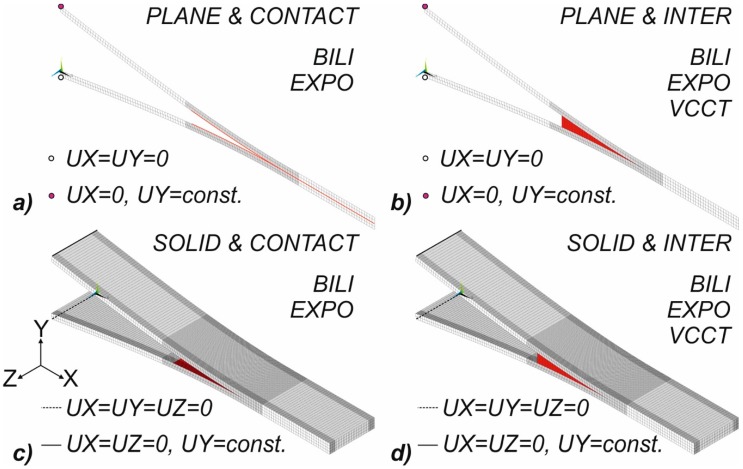
The analyzed numerical models of DCB specimens: Plane&Contact (**a**), Plane&Inter (**b**), Solid&Contact (**c**) and Solid&Inter (**d**).

Material model, geometrical model, the method of discretization, boundary conditions and the method of loads implementation are the integral elements of the numerical model. The specimen was 125-mm long, 20.0-mm wide, with two 2.08-mm-thick arms, and it had an initial crack length of 55 mm. The numerical analyses were performed in the ANSYS^®^ program based on the finite element. It should be noted that in numerical studies only the part of the sample considered in the experimental research was modeled (from the point where the load was applied to its end towards the propagation of the front of the delamination). To benchmark the numerical procedures used to reflect the DCB test, it was decided to prepare several different numerical propositions: two and three dimensional. In the case of plane models, the cohesive zones were modeled with the use of bilinear and exponential traction-separation law by application of four-node contact and inter elements (cf. Figure 4a,b). Similarly, for the 3D case, the numerical models, created by eight-node layered structural solid elements, the cohesive interfaces were modeled with the use contact and inter elements (Figure 4c,d). Additionally, for 2D and 3D models with linear interface elements (Inter), the Virtual Crack Closure Technique (VCCT) was applied (cf. Figure 4b,d). In the case of VCCT, the linear fracture criterion was implemented. The created numerical procedures are the basis for: the validation of experimental tests and the selection of appropriate discretization parameters and control parameters for geometrically nonlinear analyses. The exact denotation of the finite elements used in numerical studies is presented in Figure 3. The cohesive zones using different finite elements are marked in red in Figure 4.

To reflect the real behavior of DCB samples during numerical tests on lines (3D) or points (2D) corresponding to the piano hinge locations, the boundary conditions were assumed. In the case of three-dimensional models on the bottom line (black dashed line), the possibility of displacements in three perpendicular directions was taken (UX=UY=UZ) while on the upper line (black solid line) the possibility of displacements in the X and Z directions was taken and the constant value of displacement along the Y direction (UY=const.) was assumed. In the case of two-dimensional models, analogous boundary conditions on the upper and bottom node, with the exception of the Z direction, were adopted. With regard to the discretization carried out, it should be noted that the standard length of the finite element along the assumed crack growth direction was set at 0.25 mm. This size concerned the area before the initial delamination (5 mm) to about 40 mm behind it. Due to the presence of heterogeneity of the strain energy release rate distribution along the front of the delamination and significant decreases in its value around the free edges of the sample, in accordance with the recommendations from the authors of [27,36], two additional subareas were created around the free edges. In the central part of the sample, the size of the element along the width of the sample was set at 0.25 mm while in the areas close to the free edges, the length was equal to 0.125 mm. In the remaining area of numerical models, the size of the finite element along the length of the sample was assumed at the level of 2 mm. Moreover, the numerous numerical tests have proved that two elements along the thickness of each of the DCB sample arms is sufficient for the analysis of unidirectional laminates. If the series of data in the attached figures or the description of numerical results does not induce other information, the above discretization settings should be understood as accepted. However, if the influence of the element size on the quality of the obtained results was examined, it was limited only to the change of the length of the element along the assumed crack growth direction in the range from 5 mm before it to 40 mm behind it. In the case of two-dimensional models, analogous lengths of finite elements, for obvious reasons excluding the Z direction, were assumed. In relation to plane models, it should be highlighted that the plane strain as an element behavior and pure displacement formulation were assumed.

Except in special cases, the two-dimensional element used has two translational degrees of freedom (UX, UY) and uses the selective reduced integration method [37,38], uniform reduced integration [39], enhanced strain formulation [40,41] or simplified enhanced strain formulation [42,43]. The selective reduced integration method cannot, however, prevent any shear locking in bending-dominated problems. Despite the fact that the uniform reduced integration method helps to prevent shearing locking, the introduction of the artificial energy to control the hourglass effect diminishes solution accuracy. With regard to preliminary results, both methods generate significant differences in respect to the determined maximum forces and the corresponding displacements as well as the observed stiffness. As has been verified in some cases, the application of the uniform reduced integration method can even completely prevent convergence of numerical calculations. Both the enhanced strain formulation and the simplified enhanced strain formulation introduce four internal (user-inaccessible) degrees of freedom to overcome shear locking. All internal degrees of freedom are introduced automatically at the element level and condensed out during the solution phase of the analysis, which allows achieving satisfactory convergence with experimental results as well as with solutions resulting from the use of LEFM. The recommendations regarding the element formulations in ANSYS environment are summarized in Table 1. In the present research, the enhanced strain formulation was adopted for both 2D and 3D models. The material properties of considered material are summarized in Table 2.

Regarding the material characterization, it is worth mentioning that, although the nominal value of interfacial strength for the tested material is 39 MPa, its application to coarser meshes is useless. Hence, it was decided to analyze the considered material, also taking other traction stress values, e.g., using the Hui et al. [45] approach to the definition of the length of the cohesive zone and the number of elements in the cohesive zone suggested by Turon et al. [20].

## 5. Discussion

In the first part of the article (Mode I Interlaminar Fracture of Glass/Epoxy Unidirectional Laminates. Part I: Experimental studies), the experimental curves were compared with an analytical model using Linear Elastic Fracture Mechanics (LEFM). Due to the qualitative compatibility of obtained results, in this article, it was decided to use only analytical curves as a reference point for conducted numerical calculations. The analysis of numerical calculations was started from the comparison of results obtained using the VCCT method and the bilinear and the exponential delamination law on the examples of two-dimensional models. In addition, the investigation of the influence of the level of accepted traction stress and the size of the finite element on the global response of the DCB sample was carried out. Finally, for the determined material and discretization parameters, benchmark studies on all considered methods and numerical models (2D and 3D) were carried out and the observed differences were explained. In addition, the shape of the delamination front was analyzed and compared with the results of experimental studies.

**Figure 5:**
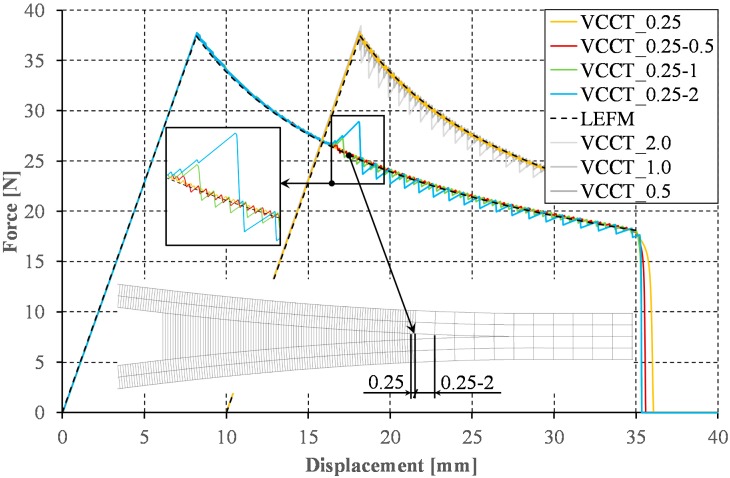
Sawtooth phenomenon for VCCT method.

**Figure 6:**
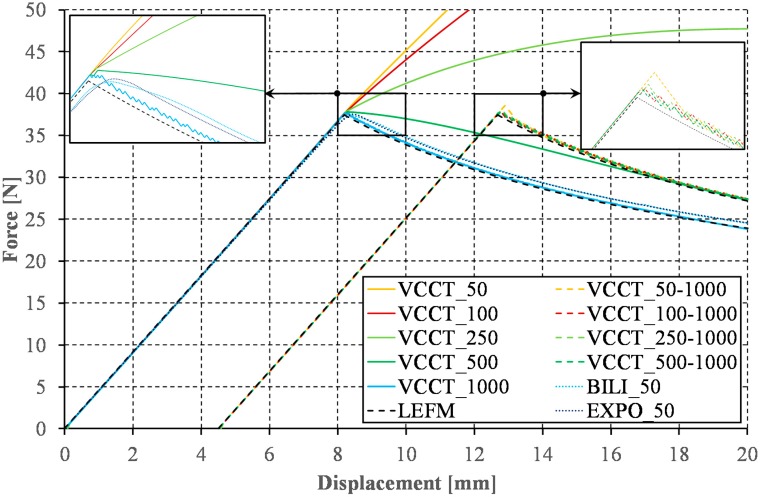
Influence of the substeps number on the load versus opening displacement curve.

**Figure 7:**
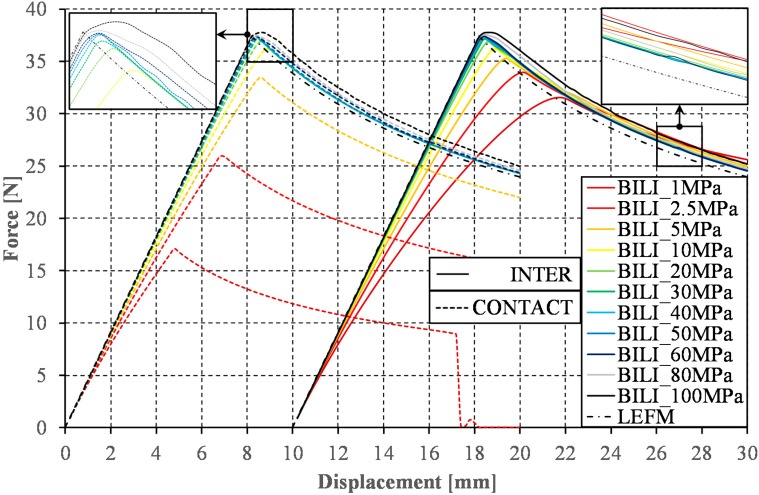
The force vs. displacement curves obtained for application of the bilinear law.

The analysis of two-dimensional numerical models was started from the VCCT method. According to the current state of knowledge and the results of numerical calculations provided, the application of the VCCT method requires the adoption a small length of the finite element in the direction of the crack propagation. According to Camanho and Hallett [46], the finite element size should be selected from the range of 1/20 < el. length/h < 1. In the analyzed case, the assumption of a finite element length at the level of 0.25 mm (the value is within the recommended range [46]) allowed obtaining a relatively smooth response (cf. Figure 5). Nevertheless, the use of smaller than the minimum recommended length of elements, excluding the increase of computation time, did not result in any adverse numerical implications (e.g., spurious oscillations). As the results of numerical calculations show, both the use of a too large finite element and the presence of any differences in finite element size along the propagation of delamination front can cause the sawtooth phenomenon, the clearer the greater the difference between the lengths of neighboring elements. The sawtooth behavior—evident in the results plotted in Figure 5—is an artifact of the VCCT implementation. The implementation of a gradual node release and ramp down of the forces at the crack tip is required to avoid this problem. It is worth mentioning that, in the data series marked as VCCT_x, x refers to the size of the finite element used to discretize the model: 5 mm before (−5) and 40 mm after (+40) the initial front of delamination, respectively. Additionally, to illustrate the effect of the change of the finite element size in the direction of crack propagation in series marked as VCCT_x-y, x and y refer to the sizes of the elements used for discretization in the ranges <−5 mm;+20 mm> and (+20 mm;+40 mm>, respectively. It should be noted that, in the following part of the article, to better visualize the obtained results, some of the curves were shifted relative to the origin of the coordinate system along the horizontal axis. These shifts correspond with: change of the element’s length in the direction of crack propagation (Figure 5), different numerical substeps in the range of the initiation and the propagation of the crack (Figure 6), division into results achieved using cohesive and contact elements (Figure 7), and division into results achieved for 2D and 3D model (Figure 9).

The small size of the finite element is not the only weakness of the VCCT method. Achieving the correct results is possible only with small time step before the crack initiation and during the crack growth. The influence of the number of applied numerical substeps in the full range of the DCB test (VCCT_50, VCCT_100, and VCCT_1000) as well as with division into two ranges of the initiation and the propagation of the crack (e.g., in the series marked as VCCT_50-1000, VCCT_50 and VCCT_1000 are the numerical substep numbers in the range of the initiation and the propagation of the crack, respectively) are compared in Figure 6. It turns out that, for the range of linear load increase, it is sufficient to implement about ten times fewer substeps than in relation to the range of nonlinear force decrease during the crack propagation (cf. Figure 6, VCCT_100-1000). The minimum increment time step allowing to achieve satisfactory results has been set at 0.001 which is a value about ten times higher than the value assumed in [47]. In addition, the curves obtained for the analogous numerical model with implemented the bilinear and the exponential cohesion law are shown in Figure 6 (series: BILI_50 and EXPO_50). It turns out that the use of a relatively small number of steps (50 in the considered cases) allows achieving qualitatively comparable results to those obtained with the implementation of the VCCT method. Despite the necessity of a small finite element size and a large number of numerical substeps assumption, the VCCT method, as no other, maintains stiffness (slope of the force–displacement curve in the linear range) to the crack initiation while in the short range before reaching the maximum applied force both the bilinear and exponential modes show a slight decrease in stiffness and nonlinear change of the applied force. The course of the curves determined with the implementation of the linear and exponential model was thoroughly analyzed.

Although in the analyzed case the VCCT method requires only the definition of the Mode I interlaminar fracture toughness, which can be directly determined from, e.g., ASTM standard [48], the necessity to implement the shape of the initial crack front and high computational cost make this method unsuitable for many modern problems [44]. In turn, the popularity of the cohesive zone method is still growing. Regardless of the implemented law of delamination, this method, besides the declaration of G_1C_ value, requires the definition of the maximal traction stress. Although there are methods for the experimental determination of the traction stress values [49], in the present study, it was decided to examine the influence of the interfacial strength on the nature of the global response recorded during the DCB test. In numerical calculations, values of the traction stresses in the range from 1 MPa to 100 MPa were adopted (cf. Figure 7). This range includes has the interfacial strength of most contemporary composite materials [20]. The interfacial strength of most modern composite materials is within this range [49]. From the analyses carried out with the element length equal to 0.025 mm, it can be noticed that the relative convergence of results was obtained for traction stresses from the range of 20–60 MPa for both the contact cohesive zone model (CONTACT) and for the cohesive elements (INTER). In the range of the crack propagation, the determined curves find their course slightly above the reference curves (LEFM). This effect is slightly smaller when using contact elements than in the case of INTER type elements. The declaration of higher values of the traction stresses results in the overestimation of the maximum applied load during DCB test (F_max_). In addition, the course of the curve in the crack propagation range is over the other determined curves. The traction stress acceptance in the range from 1 to 10 MPa significantly underestimates the maximum force (F_max_) as well as the character of the force vs. displacement curves, although using cohesive elements, these paths converge at a later stage with all determined curves. The qualitative differences in the results achieved between models with implemented INTER and CONTACT elements in the range of traction stresses from 1 to 10 MPa can be astonishing. It is reported, however, that this problem has been resolved since version ANSYS 18.2. Nevertheless, in older versions of the applied software, the use of INTER elements coupled with the application of the low traction stress for a small element may lead to an underestimation of the maximum opening force and qualitative difference in the global response recorded during the DCB test. It is recommended that, when using a small element size (<0.1 mm), a transverse tensile strength should be used as the reference traction stress value.

**Figure 8:**
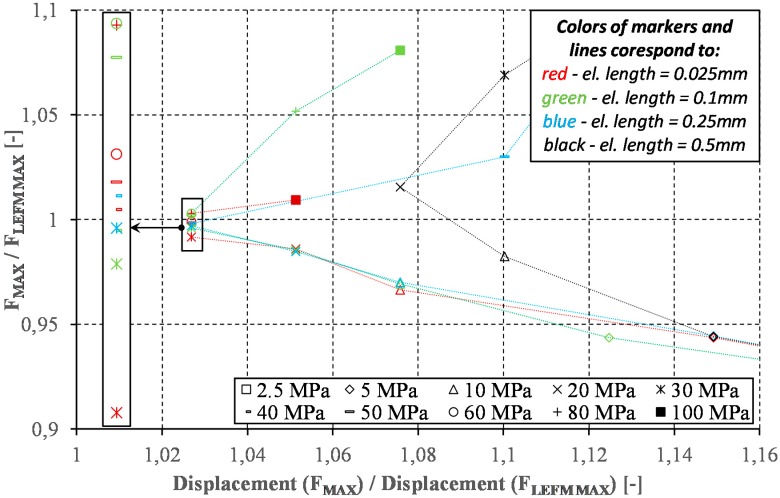
Influence of the finite element size and the values of the traction stress on the solution’s compatibility with the analytical model (LEFM).

Assuming the Hui et al. model [45] for the applied finite element length (0.025 mm) and following the recommendations of Turon et al. [20] that the cohesion zone should be represented by 5–8 finite elements, the theoretical value of maximum stresses from 58.6 MPa to 74.2 MPa can be determined. It is not surprising, therefore, that the assumption of higher traction stresses leads to the described overestimation of the results, because the cohesion zone at maximum traction stresses higher than 74.2 MPa is not represented by a sufficient number of finite elements. Numerical studies have proven that the implementation of the interfacial strength, determined directly from experimental research, to numerical models characterized by coarser mesh does not allow achieving convergent results. In the next step, it was decided to examine the effect of changes in traction stress values in relation to different finite element lengths (cf. Figure 8).

Taking as a reference points of the analytical values of the maximum applied load during DCB test (*F_MAX LEFM_*) and the corresponding displacement (*Displacement(F_MAX LEFM_)*), the results obtained using the bilinear model for different maximum values of the traction stresses and different finite element lengths along the crack growth were compiled. The first observation of the obtained results is the fact that the application of the bilinear model may, at certain values of assumed traction stresses, lead to almost identical results with respect to the referential maximum force but never maintaining the same as the analytical value of displacement. The best achieved *Displacement(F_MAX_)* to *Displacement(F_MAX LEFM_)* ratio is about 1.03, which corresponds to the differences in stiffness (slope of the force–displacement curves) observed in the range of nonlinear force increase (Figure 8). The most important observation, however, concerns the acceptable level of traction stress in order to correctly reflect the behavior of the cohesive zone. The acceptable convergence level for the element length of 0.025 mm (see zoomed area of the black rectangle in Figure 8) is achieved for traction stresses from 30 to 80 MPa which translates, assuming the Hui et al. [45] model, into the representation of a cohesive zone from 30 to 5 elements. For an element with a length of 0.1 mm, a similar effect is achieved for the traction stresses in the range from 30 to 60 MPa, which translates into a representation of the cohesion zone from 2 to 8 elements. As the size of the element increases, the range of acceptable traction stresses decreases so that in the case of a 0.25 mm element, acceptable results are achieved among the considered values by 30 and 40 MPa, representing the cohesion zone only by two or three elements. The declaration of finite element length equal to 0.5 mm results in the best solution to finding traction stress at the level of 20 MPa, which overestimates the maximum force registered in the DCB test and the corresponding displacement by nearly 2% and 8%, respectively. This corresponds to the representation of the cohesive zone by seven elements.

**Figure 9:**
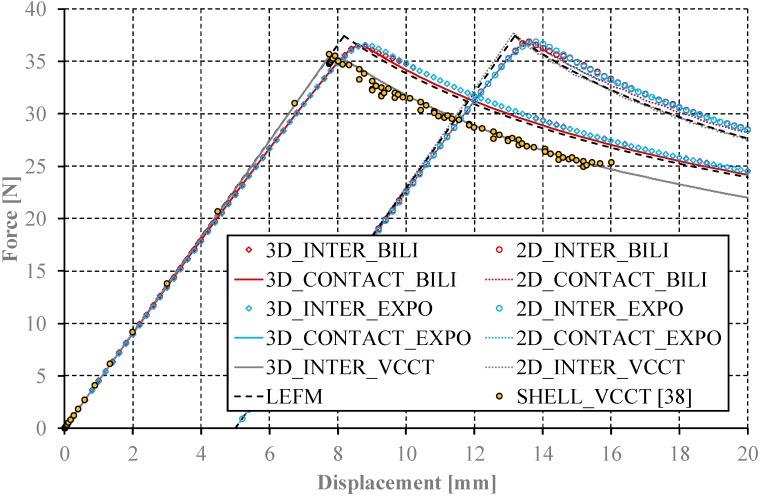
Comparison of the investigated models.

To eliminate the influence of finite element size, it was decided to conduct benchmark studies of all considered models assuming the element length of 0.25 mm along the crack growth direction and the traction stress of 20 MPa, reflecting the representation of the cohesive zone by eight elements (cf. Figure 9). The comparative research shows that in the two-dimensional approach all analyzed methods (VCCT, CZM: BILI and EXPO) allow achieving a high compliance in relation to the reference results. The analysis of the determined curves shows that the results obtained with the implementation of the cohesive elements slightly exceed those determined from the models using contact elements. Excluding the results determined with the implementation of the VCCT method, all the above statements are identical also for the behavior of three-dimensional models. Although the VCCT method in two-dimensional approach is characterized by the best compliance in relation to the adopted analytical model, its implementation to the three-dimensional model does not lead to identical results. To validate this observation, several additional tests were carried out. First, numerical calculations were performed with smaller element length along the crack growth direction of (0.125 mm and 0.0625 mm). In addition, the results obtained using the shell model (SHELL_VCCT) were enclosed, the correctness of which has been well documented in international literature [36], but not yet combined with the results of numerical research using two- and three-dimensional models with bilinear and exponential cohesion law. Both approaches resulted in identical results. It turns out that the implementation of the VCCT method in the three-dimensional problem leads to underestimation of the maximum load during DCB test and the corresponding displacement. In addition, the course of the determined curve during the crack growth is contained entirely under the analytical curve and is parallel to analytical solution. Differences in the determined forces for subsequent displacement values are approximately 2.2 N.

**Figure 10:**
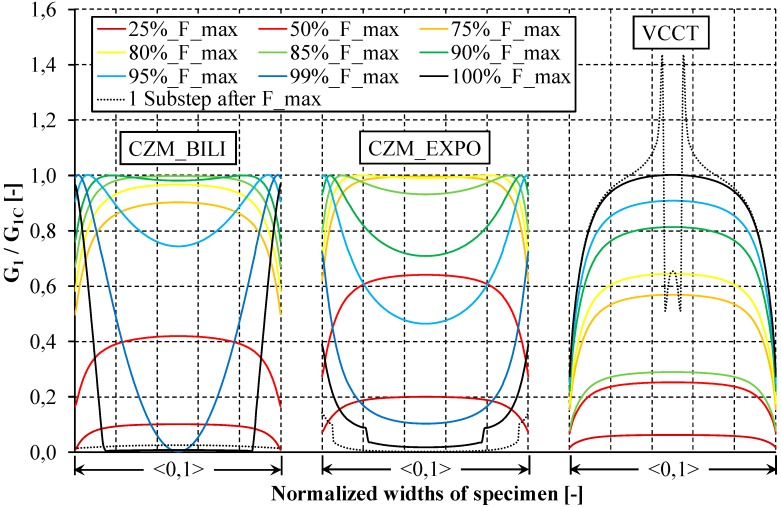
Dimensionless distribution of the SERR along the initial delamination front.

To investigate the reason for the differences between the analyzed numerical models, the distribution of the energy release rate along the width of the DCB sample was thoroughly analyzed. The determined dimensionless distributions of the strain energy release rate (G_I_/G_IC_) along the initial delamination front for different load values are provided in Figure 10. Analyzing the obtained curves, it can be seen that in the initial load range, the numerical distribution of the energy release rate (up to 50% of the maximum applied load during DCB test–50%_F_max) in all analyzed models (VCCT, CZM_BILI, and CZM_EXPO) has a similar nonlinear course with maximum values achieved in the middle of the sample width. Nevertheless, in the case of the bilinear and exponential models, after exceeding about 75–80% of the maximum applied in the numerical DCB test, the strain energy release rates assume maximum values on the width of approximately 60% of the sample. Further loading process leads in numerical approach to the increase of the energy release rate in the remaining part of the initial crack front (propagates to free edges) while in the central part of the specimen there is a decrease of the SERR caused by exceeding the maximum assumed traction stresses on the initial crack front and propagation of the delamination front. As a consequence, there is almost a complete reduction of the strain energy release rate over the entire width of the initial crack front line. It turns out that reaching the maximum load during the DCB test corresponds to almost zero distribution of the SERR along the initial delamination front. The changes observed with the implementation of the VCCT method have a different character. In this case, the distribution of the SERR along the initial crack front is unchanging until the maximum force is reached. When the critical energy release rate value is exceeded in about 10% of the central part of the sample, the force determined in the DCB test reaches its maximum value. This results in a sudden change in the SERR distribution. As in the case of the bilinear and exponential model, the decrease of the SERR value propagates towards the free edges of the sample. The main difference between the presented distributions of the SERR lies in the fact that in the case of the CZM models, the maximum force is achieved in the absence of almost any traction on the initial delamination front while in the VCCT method in 90% of the initial crack front the values of determined SERR are still below the critical ones. Noteworthy is the fact that using the bilinear and exponential model at no stage of the numerical calculation and at any node, the critical strain energy release rate was exceeded while the excess of the critical SERR in the VCCT method at the initiation of the crack propagation can reach up to 50%. It should be noted that the included distributions refer to the SERR values achieved only on the initial crack front line. They should not be interpreted as a shape of the propagating delamination front.

**Figure 11:**
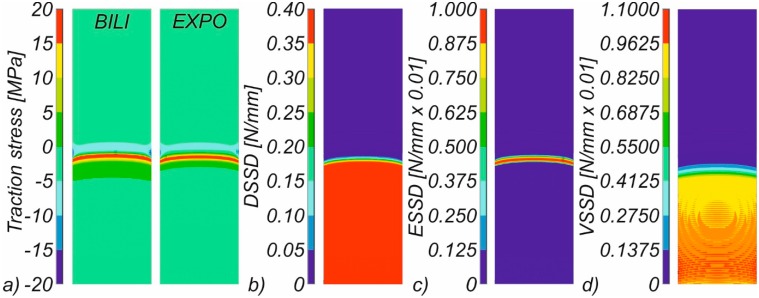
Contour maps of: traction stress (**a**); damage strain energy density (**b**); elastic strain energy density (**c**); and visco-regularization strain energy density (**d**).

Despite the slight differences in the SERR distributions determined for the bilinear and exponential model, the contour maps of the traction stresses (determined for the opening displacement of 20 mm) allow finally concluding that both delamination laws remain in mutual correlation (Figure 11a). Regarding the shape of the propagation front, it can be noticed that, in all analyzed numerical cases, the SERR distribution had the symmetric character in relation to the sample’s symmetry plane. Energies had the lowest values at the edges of the sample and relatively a comparable value of G_I_ energy for nodes located at the distances greater than 2.5 mm from the edges of the analyzed samples (cf. Figure 11b). This observation is in line with the results reported by Kaushik and Ghosh [50]. Using the bilinear model, one can also see the front of elastic strain energy formed in front of the propagating crack (Figure 11c). In all analyses carried out with the implementation of the CZM, the viscous regularization was used to stabilize interface delamination and avoid convergence difficulties. In all conducted analyses, the value of the fictitious viscosity was assumed as equal to 0.1 × (1/Substep number). The distribution of the visco-regularization strain energy density is included in Figure 11d.

**Figure 12:**
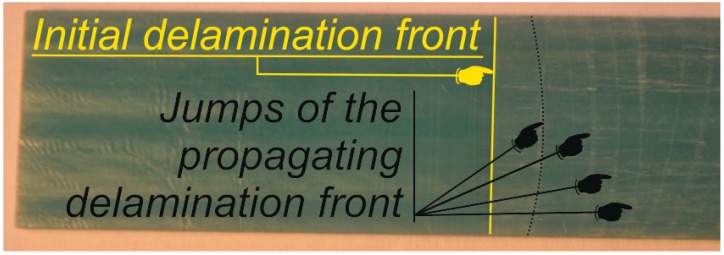
Scratches left by unstable crack propagation during DCB test.

Scratches left on the crack propagation front (Figure 12) correspond with the load drops recorded during the experimental DCB tests (cf. Figure 3 in Part I: Experimental studies). Most often, as the front of the crack propagates, the effect of sudden load drops disappears due to the presence of the fiber bridging phenomenon. Nevertheless, in the initial phase of propagation, the scratches left on the crack front reliably reflect both the distribution of the traction stresses as well as the damage and elastic strain energy density observed in the frame of numerical studies. Scratches left by the propagating front of the delamination correspond with the results of the ultrasonic C-scan image of DCB specimen after fatigue precracking obtained by Czabaj and Ratcliffe [25].

## 6. Conclusions

Within the present study, numerical investigations of the DCB specimen were conducted. The considered material was GFRP laminate. Benchmark studies of two- and three-dimensional DCB models with implementation of contact and cohesive elements were performed. The crack growth phenomenon was modeled using the Virtual Crack Closure Technique (VCCT) and the CZM method with the implementation of the bilinear and the exponential delamination law. Based on the performed numerical studies, it has been concluded that:

- Excluding the results determined with the implementation of the VCCT method for the 3D case, the benchmark studies show that in the two- and three-dimensional approaches of the CZM and the VCCT allow achievieng a high compliance in relation to the reference results.
- The application of the VCCT in the three-dimensional model leads to an underestimation of the maximum applied load during DCB test.
- In the case of the CZM models, the maximum force (F_max_) is achieved in the absence of almost any traction on the initial delamination front while, in the VCCT, in 90% of the initial crack front, the SERRs are still below the critical values.
- Using the bilinear and exponential model at no stage of the numerical calculation and at any node, the critical strain energy release rate was exceeded while the excess of the critical SERR in the VCCT method at the initiation of the crack propagation can reach up to 50%.
- The scratches left on the experimental crack front correspond to both the distribution of the traction stresses as well as the damage and elastic strain energy density observed in the numerical studies.
- 2D models, due to the short CPU time, are perfect for estimating the minimum length of the element along the propagation direction of the delamination front and validation of the assumed values of traction stresses. These results can be successfully implemented into three-dimensional models.
- The adoption of the characteristic length of the cohesive zone in accordance with the Hui model and the representation of the cohesive zone by five elements led to satisfactory results.
- Current implementation of the VCCT method in the ANSYS software allows for a quasi-static propagation analysis, however, the stair-stepped delamination front caused by the simple node release approach remains an issue (sawtooth phenomenon).
- In bending-dominated problems, enhanced or simplified enhanced strain formulation should be used.
- The superiority of the CZM method over the VCCT has been confirmed by: (i) the possibility of applying the method for coarse meshes; (ii) convergence of results achieved for both two- and three-dimensional models; (iii) significantly shorter CPU time; and (iv) no need of the initial delamination front assumption.

## Figures and Tables

**Table 1:** Recommendation of the element formulations.

Element Technology	2-D Models	3-D Models
Selective reduced integration method	No	N/A
Uniform reduced integration with hourglass control	No	N/A
Enhanced strain formulation	Yes	Yes
Simplified enhanced strain formulation	Yes	Yes
N/A, not available in the case of layered structural solid elements		

**Table 2:** Mechanical and interface material properties of GFRP material [44].

E_11_	E_22_ = E_33_	G_12_ = G_13_	G_23_	ν_12_ = ν_13_	ν_23_	G_IC_	τ_3_
38.5 GPa	8.1 GPa	2.6 GPa	1.9 GPa	0.27	0.34	0.34 N/mm	39 MPa
